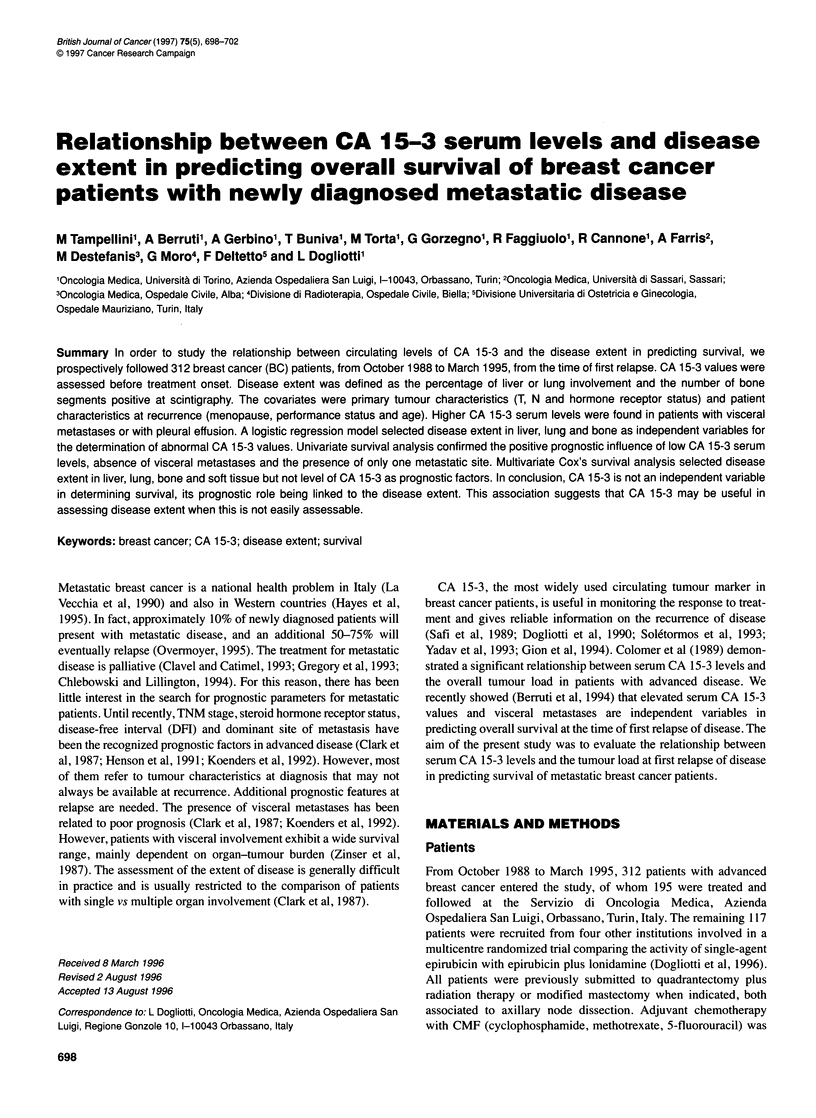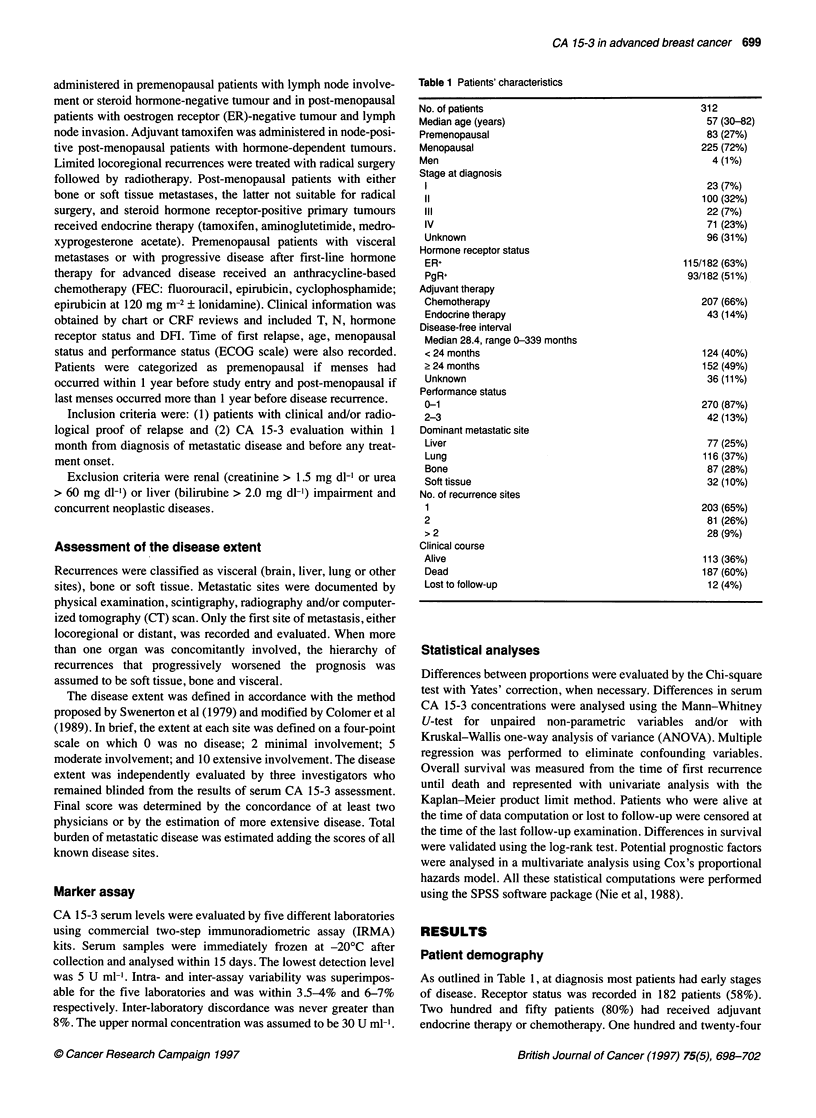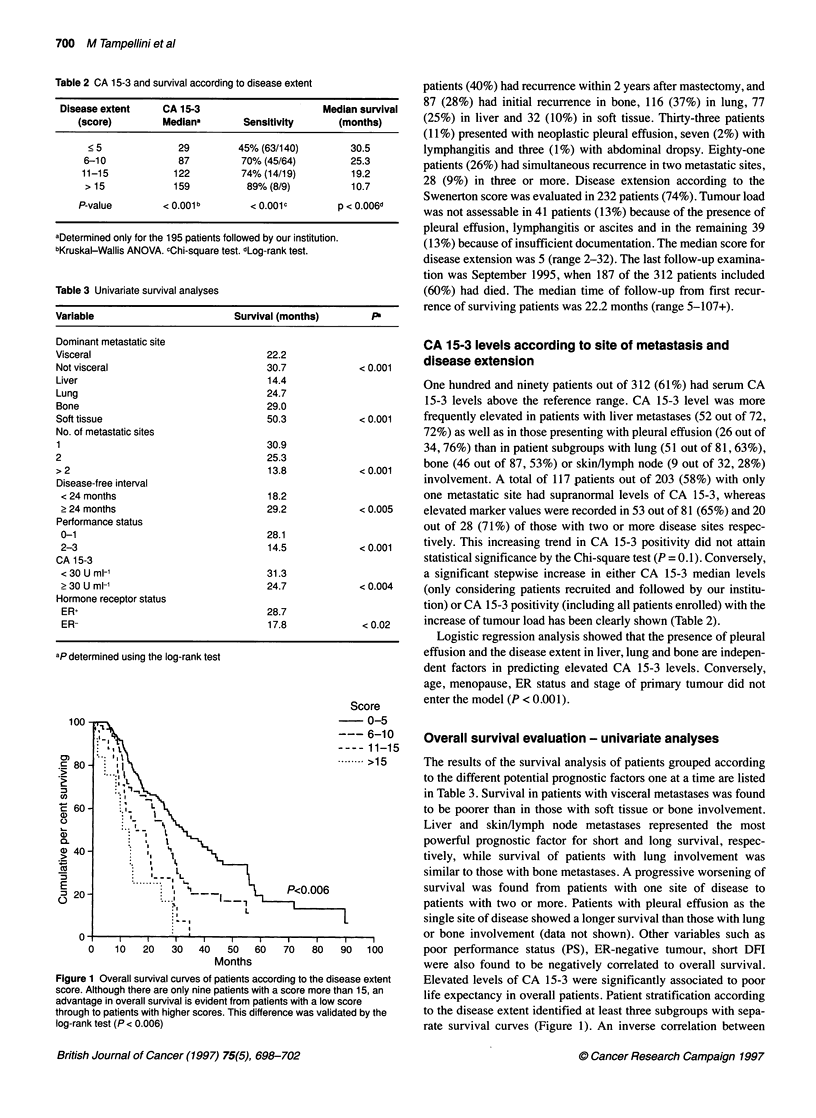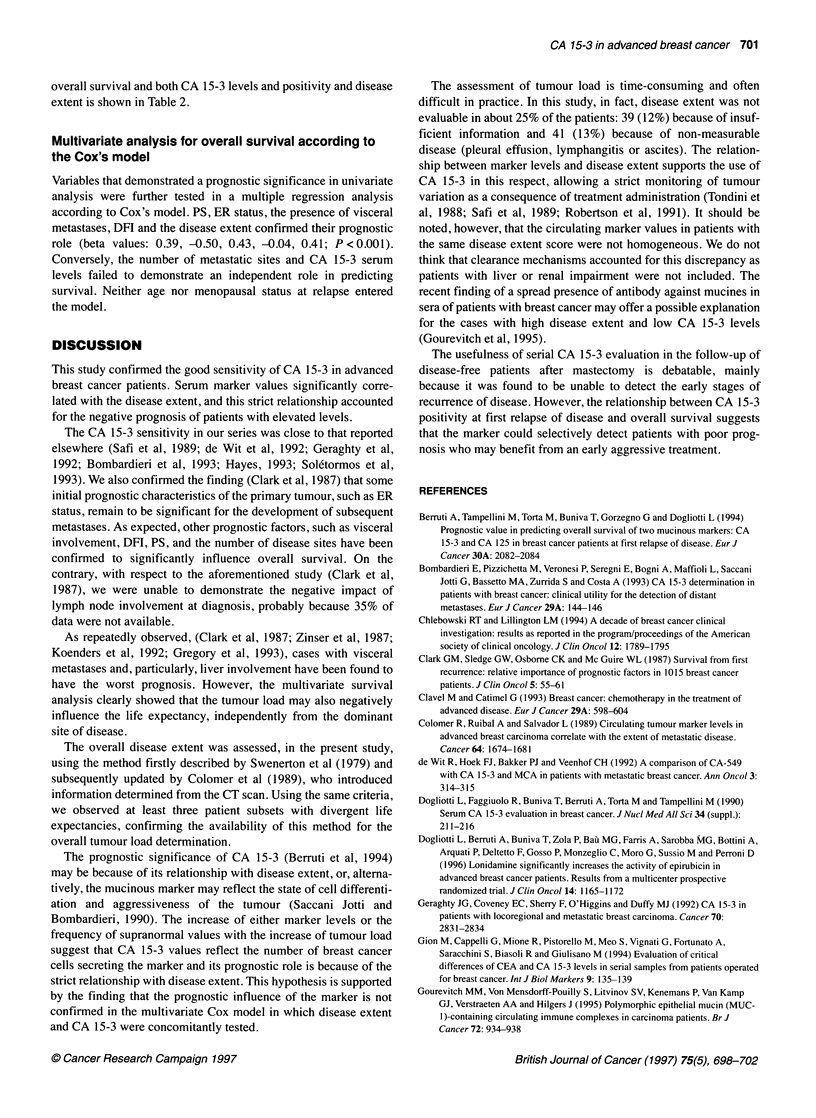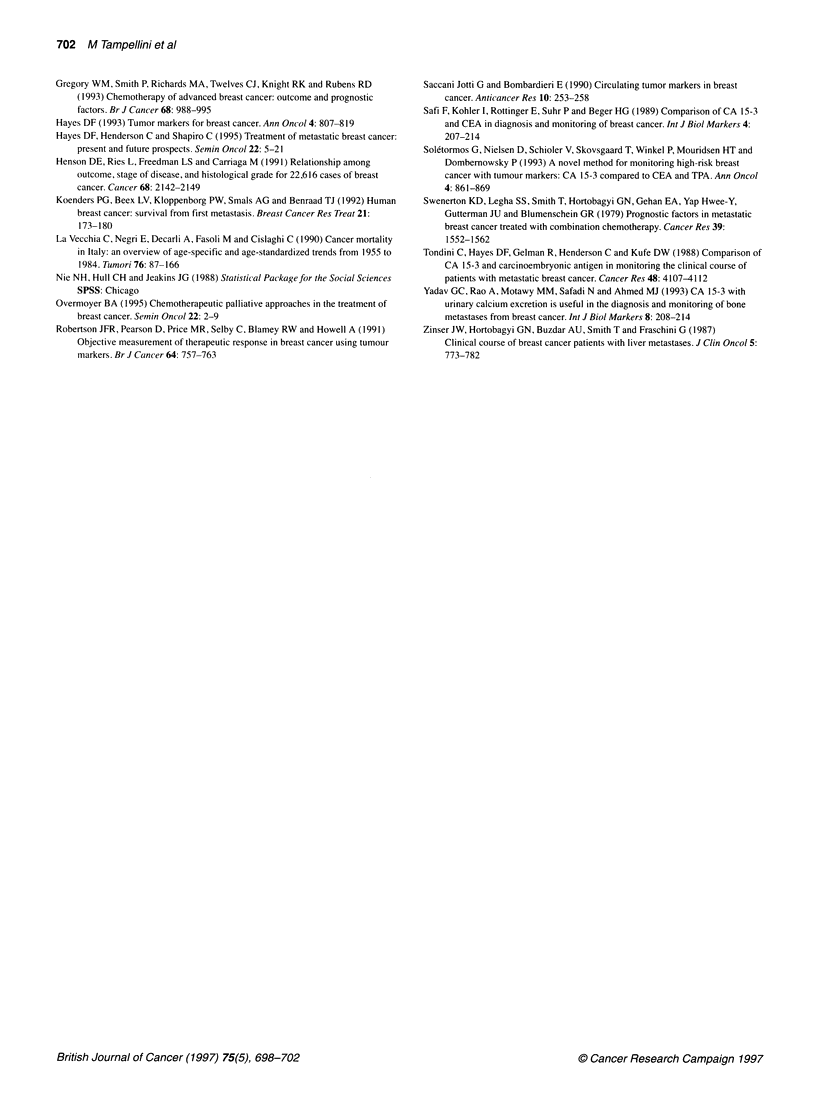# Relationship between CA 15-3 serum levels and disease extent in predicting overall survival of breast cancer patients with newly diagnosed metastatic disease.

**DOI:** 10.1038/bjc.1997.124

**Published:** 1997

**Authors:** M. Tampellini, A. Berruti, A. Gerbino, T. Buniva, M. Torta, G. Gorzegno, R. Faggiuolo, R. Cannone, A. Farris, M. Destefanis, G. Moro, F. Deltetto, L. Dogliotti

**Affiliations:** Oncologia Medica, Università di Torino, Azienda Ospedaliera San Luigi,Turin, Italy.

## Abstract

In order to study the relationship between circulating levels of CA 15-3 and the disease extent in predicting survival, we prospectively followed 312 breast cancer (BC) patients, from October 1988 to March 1995, from the time of first relapse. CA 15-3 values were assessed before treatment onset. Disease extent was defined as the percentage of liver or lung involvement and the number of bone segments positive at scintigraphy. The covariates were primary tumour characteristics (T, N and hormone receptor status) and patient characteristics at recurrence (menopause, performance status and age). Higher CA 15-3 serum levels were found in patients with visceral metastases or with pleural effusion. A logistic regression model selected disease extent in liver, lung and bone as independent variables for the determination of abnormal CA 15-3 values. Univariate survival analysis confirmed the positive prognostic influence of low CA 15-3 serum levels, absence of visceral metastases and the presence of only one metastatic site. Multivariate Cox's survival analysis selected disease extent in liver, lung, bone and soft tissue but not level of CA 15-3 as prognostic factors. In conclusion, CA 15-3 is not an independent variable in determining survival, its prognostic role being linked to the disease extent. This association suggests that CA 15-3 may be useful in assessing disease extent when this is not easily assessable.


British Journal of Cancer (1997) 75(5), 698–702
© 1997 Cancer Research Campaign

### Relationship between CA 15-3 serum levels and disease extent in predicting overall survival of breast cancer patients with newly diagnosed metastatic disease

**M Tampellini[1], A Berruti[1], A Gerbino[1], T Buniva[1], M Torta[1], G Gorzegno[1], R Faggiuolo[1], R Cannone[1], A Farris[2], M Destefanis[3], G Moro[4], F Deltetto[5] and L Dogliotti[1]**

[1]Oncologia Medica, Università di Torino, Azienda Ospedaliera San Luigi, I-10043, Orbassano, Turin; [2]Oncologia Medica, Università di Sassari, Sassari; [3]Oncologia Medica, Ospedale Civile, Alba; [4]Divisione di Radioterapia, Ospedale Civile, Biella; [5]Divisione Universitaria di Ostetricia e Ginecologia, Ospedale Mauriziano, Turin, Italy

**Summary** In order to study the relationship between circulating levels of CA 15-3 and the disease extent in predicting survival, we prospectively followed 312 breast cancer (BC) patients, from October 1988 to March 1995, from the time of first relapse. CA 15-3 values were assessed before treatment onset. Disease extent was defined as the percentage of liver or lung involvement and the number of bone segments positive at scintigraphy. The covariates were primary tumour characteristics (T, N and hormone receptor status) and patient characteristics at recurrence (menopause, performance status and age). Higher CA 15-3 serum levels were found in patients with visceral metastases or with pleural effusion. A logistic regression model selected disease extent in liver, lung and bone as independent variables for the determination of abnormal CA 15-3 values. Univariate survival analysis confirmed the positive prognostic influence of low CA 15-3 serum levels, absence of visceral metastases and the presence of only one metastatic site. Multivariate Cox's survival analysis selected disease extent in liver, lung, bone and soft tissue but not level of CA 15-3 as prognostic factors. In conclusion, CA 15-3 is not an independent variable in determining survival, its prognostic role being linked to the disease extent. This association suggests that CA 15-3 may be useful in assessing disease extent when this is not easily assessable.

**Keywords:** breast cancer; CA 15-3; disease extent; survival

Metastatic breast cancer is a national health problem in Italy (La Vecchia et al, 1990) and also in Western countries (Hayes et al, 1995). In fact, approximately 10% of newly diagnosed patients will present with metastatic disease, and an additional 50–75% will eventually relapse (Overmoyer, 1995). The treatment for metastatic disease is palliative (Clavel and Catimel, 1993; Gregory et al, 1993; Chlebowski and Lillington, 1994). For this reason, there has been little interest in the search for prognostic parameters for metastatic patients. Until recently, TNM stage, steroid hormone receptor status, disease-free interval (DFI) and dominant site of metastasis have been the recognized prognostic factors in advanced disease (Clark et al, 1987; Henson et al, 1991; Koenders et al, 1992). However, most of them refer to tumour characteristics at diagnosis that may not always be available at recurrence. Additional prognostic features at relapse are needed. The presence of visceral metastases has been related to poor prognosis (Clark et al, 1987; Koenders et al, 1992). However, patients with visceral involvement exhibit a wide survival range, mainly dependent on organ–tumour burden (Zinser et al, 1987). The assessment of the extent of disease is generally difficult in practice and is usually restricted to the comparison of patients with single *vs* multiple organ involvement (Clark et al, 1987).

*Received 8 March 1996*
*Revised 2 August 1996*
*Accepted 13 August 1996*

*Correspondence to:* L Dogliotti, Oncologia Medica, Azienda Ospedaliera San Luigi, Regione Gonzole 10, I-10043 Orbassano, Italy

CA 15-3, the most widely used circulating tumour marker in breast cancer patients, is useful in monitoring the response to treatment and gives reliable information on the recurrence of disease (Safi et al, 1989; Dogliotti et al, 1990; Solétormos et al, 1993; Yadav et al, 1993; Gion et al, 1994). Colomer et al (1989) demonstrated a significant relationship between serum CA 15-3 levels and the overall tumour load in patients with advanced disease. We recently showed (Berruti et al, 1994) that elevated serum CA 15-3 values and visceral metastases are independent variables in predicting overall survival at the time of first relapse of disease. The aim of the present study was to evaluate the relationship between serum CA 15-3 levels and the tumour load at first relapse of disease in predicting survival of metastatic breast cancer patients.

### MATERIALS AND METHODS

### Patients

From October 1988 to March 1995, 312 patients with advanced breast cancer entered the study, of whom 195 were treated and followed at the Servizio di Oncologia Medica, Azienda Ospedaliera San Luigi, Orbassano, Turin, Italy. The remaining 117 patients were recruited from four other institutions involved in a multicentre randomized trial comparing the activity of single-agent epirubicin with epirubicin plus lonidamine (Dogliotti et al, 1996). All patients were previously submitted to quadrantectomy plus radiation therapy or modified mastectomy when indicated, both associated to axillary node dissection. Adjuvant chemotherapy with CMF (cyclophosphamide, methotrexate, 5-fluorouracil) was administered in premenopausal patients with lymph node involvement or steroid hormone-negative tumour and in post-menopausal patients with oestrogen receptor (ER)-negative tumour and lymph node invasion. Adjuvant tamoxifen was administered in node-positive post-menopausal patients with hormone-dependent tumours. Limited locoregional recurrences were treated with radical surgery followed by radiotherapy. Post-menopausal patients with either bone or soft tissue metastases, the latter not suitable for radical surgery, and steroid hormone receptor-positive primary tumours received endocrine therapy (tamoxifen, aminoglutetimide, medroxyprogesterone acetate). Premenopausal patients with visceral metastases or with progressive disease after first-line hormone therapy for advanced disease received an anthracycline-based chemotherapy (FEC: fluorouracil, epirubicin, cyclophosphamide; epirubicin at 120 mg $m^{-2}$ ± lonidamine). Clinical information was obtained by chart or CRF reviews and included T, N, hormone receptor status and DFI. Time of first relapse, age, menopausal status and performance status (ECOG scale) were also recorded. Patients were categorized as premenopausal if menses had occurred within 1 year before study entry and post-menopausal if last menses occurred more than 1 year before disease recurrence.

Inclusion criteria were: (1) patients with clinical and/or radiological proof of relapse and (2) CA 15-3 evaluation within 1 month from diagnosis of metastatic disease and before any treatment onset.

Exclusion criteria were renal (creatinine > 1.5 mg $dl^{-1}$ or urea > 60 mg $dl^{-1}$) or liver (bilirubine > 2.0 mg $dl^{-1}$) impairment and concurrent neoplastic diseases.

### Assessment of the disease extent

Recurrences were classified as visceral (brain, liver, lung or other sites), bone or soft tissue. Metastatic sites were documented by physical examination, scintigraphy, radiography and/or computerized tomography (CT) scan. Only the first site of metastasis, either locoregional or distant, was recorded and evaluated. When more than one organ was concomitantly involved, the hierarchy of recurrences that progressively worsened the prognosis was assumed to be soft tissue, bone and visceral.

The disease extent was defined in accordance with the method proposed by Swenerton et al (1979) and modified by Colomer et al (1989). In brief, the extent at each site was defined on a four-point scale on which 0 was no disease; 2 minimal involvement; 5 moderate involvement; and 10 extensive involvement. The disease extent was independently evaluated by three investigators who remained blinded from the results of serum CA 15-3 assessment. Final score was determined by the concordance of at least two physicians or by the estimation of more extensive disease. Total burden of metastatic disease was estimated adding the scores of all known disease sites.

### Marker assay

CA 15-3 serum levels were evaluated by five different laboratories using commercial two-step immunoradiometric assay (IRMA) kits. Serum samples were immediately frozen at –20°C after collection and analysed within 15 days. The lowest detection level was 5 U $ml^{-1}$. Intra- and inter-assay variability was superimposable for the five laboratories and was within 3.5–4% and 6–7% respectively. Inter-laboratory discordance was never greater than 8%. The upper normal concentration was assumed to be 30 U $ml^{-1}$.

**Table 1** Patients' characteristics

| | |
|---|---|
| No. of patients | 312 |
| Median age (years) | 57 (30–82) |
| Premenopausal | 83 (27%) |
| Menopausal | 225 (72%) |
| Men | 4 (1%) |
| Stage at diagnosis | |
| I | 23 (7%) |
| II | 100 (32%) |
| III | 22 (7%) |
| IV | 71 (23%) |
| Unknown | 96 (31%) |
| Hormone receptor status | |
| $ER^+$ | 115/182 (63%) |
| $PgR^+$ | 93/182 (51%) |
| Adjuvant therapy | |
| Chemotherapy | 207 (66%) |
| Endocrine therapy | 43 (14%) |
| Disease-free interval | |
| Median 28.4, range 0–339 months | |
| < 24 months | 124 (40%) |
| ≥ 24 months | 152 (49%) |
| Unknown | 36 (11%) |
| Performance status | |
| 0–1 | 270 (87%) |
| 2–3 | 42 (13%) |
| Dominant metastatic site | |
| Liver | 77 (25%) |
| Lung | 116 (37%) |
| Bone | 87 (28%) |
| Soft tissue | 32 (10%) |
| No. of recurrence sites | |
| 1 | 203 (65%) |
| 2 | 81 (26%) |
| > 2 | 28 (9%) |
| Clinical course | |
| Alive | 113 (36%) |
| Dead | 187 (60%) |
| Lost to follow-up | 12 (4%) |

### Statistical analyses

Differences between proportions were evaluated by the Chi-square test with Yates' correction, when necessary. Differences in serum CA 15-3 concentrations were analysed using the Mann–Whitney *U*-test for unpaired non-parametric variables and/or with Kruskal–Wallis one-way analysis of variance (ANOVA). Multiple regression was performed to eliminate confounding variables. Overall survival was measured from the time of first recurrence until death and represented with univariate analysis with the Kaplan–Meier product limit method. Patients who were alive at the time of data computation or lost to follow-up were censored at the time of the last follow-up examination. Differences in survival were validated using the log-rank test. Potential prognostic factors were analysed in a multivariate analysis using Cox's proportional hazards model. All these statistical computations were performed using the SPSS software package (Nie et al, 1988).

### RESULTS

### Patient demography

As outlined in Table 1, at diagnosis most patients had early stages of disease. Receptor status was recorded in 182 patients (58%). Two hundred and fifty patients (80%) had received adjuvant endocrine therapy or chemotherapy. One hundred and twenty-four

© Cancer Research Campaign 1997

Table 2 CA 15-3 and survival according to disease extent

| Disease extent (score) | CA 15-3 Median[a] | Sensitivity | Median survival (months) |
|---|---|---|---|
| ≤ 5 | 29 | 45% (63/140) | 30.5 |
| 6–10 | 87 | 70% (45/64) | 25.3 |
| 11–15 | 122 | 74% (14/19) | 19.2 |
| > 15 | 159 | 89% (8/9) | 10.7 |
| *P*-value | < 0.001[b] | < 0.001[c] | p < 0.006[d] |

[a]Determined only for the 195 patients followed by our institution.
[b]Kruskal–Wallis ANOVA. [c]Chi-square test. [d]Log-rank test.

Table 3 Univariate survival analyses

| Variable | Survival (months) | P[a] |
|---|---|---|
| Dominant metastatic site | | |
| Visceral | 22.2 | |
| Not visceral | 30.7 | < 0.001 |
| Liver | 14.4 | |
| Lung | 24.7 | |
| Bone | 29.0 | |
| Soft tissue | 50.3 | < 0.001 |
| No. of metastatic sites | | |
| 1 | 30.9 | |
| 2 | 25.3 | |
| > 2 | 13.8 | < 0.001 |
| Disease-free interval | | |
| < 24 months | 18.2 | |
| ≥ 24 months | 29.2 | < 0.005 |
| Performance status | | |
| 0–1 | 28.1 | |
| 2–3 | 14.5 | < 0.001 |
| CA 15-3 | | |
| < 30 U ml⁻¹ | 31.3 | |
| ≥ 30 U ml⁻¹ | 24.7 | < 0.004 |
| Hormone receptor status | | |
| ER+ | 28.7 | |
| ER− | 17.8 | < 0.02 |

[a]*P* determined using the log-rank test

Figure 1 Overall survival curves of patients according to the disease extent score. Although there are only nine patients with a score more than 15, an advantage in overall survival is evident from patients with a low score through to patients with higher scores. This difference was validated by the log-rank test (*P* < 0.006)

patients (40%) had recurrence within 2 years after mastectomy, and 87 (28%) had initial recurrence in bone, 116 (37%) in lung, 77 (25%) in liver and 32 (10%) in soft tissue. Thirty-three patients (11%) presented with neoplastic pleural effusion, seven (2%) with lymphangitis and three (1%) with abdominal dropsy. Eighty-one patients (26%) had simultaneous recurrence in two metastatic sites, 28 (9%) in three or more. Disease extension according to the Swenerton score was evaluated in 232 patients (74%). Tumour load was not assessable in 41 patients (13%) because of the presence of pleural effusion, lymphangitis or ascites and in the remaining 39 (13%) because of insufficient documentation. The median score for disease extension was 5 (range 2–32). The last follow-up examination was September 1995, when 187 of the 312 patients included (60%) had died. The median time of follow-up from first recurrence of surviving patients was 22.2 months (range 5–107+).

### CA 15-3 levels according to site of metastasis and disease extension

One hundred and ninety patients out of 312 (61%) had serum CA 15-3 levels above the reference range. CA 15-3 level was more frequently elevated in patients with liver metastases (52 out of 72, 72%) as well as in those presenting with pleural effusion (26 out of 34, 76%) than in patient subgroups with lung (51 out of 81, 63%), bone (46 out of 87, 53%) or skin/lymph node (9 out of 32, 28%) involvement. A total of 117 patients out of 203 (58%) with only one metastatic site had supranormal levels of CA 15-3, whereas elevated marker values were recorded in 53 out of 81 (65%) and 20 out of 28 (71%) of those with two or more disease sites respectively. This increasing trend in CA 15-3 positivity did not attain statistical significance by the Chi-square test ($P = 0.1$). Conversely, a significant stepwise increase in either CA 15-3 median levels (only considering patients recruited and followed by our institution) or CA 15-3 positivity (including all patients enrolled) with the increase of tumour load has been clearly shown (Table 2).

Logistic regression analysis showed that the presence of pleural effusion and the disease extent in liver, lung and bone are independent factors in predicting elevated CA 15-3 levels. Conversely, age, menopause, ER status and stage of primary tumour did not enter the model ($P < 0.001$).

### Overall survival evaluation – univariate analyses

The results of the survival analysis of patients grouped according to the different potential prognostic factors one at a time are listed in Table 3. Survival in patients with visceral metastases was found to be poorer than in those with soft tissue or bone involvement. Liver and skin/lymph node metastases represented the most powerful prognostic factor for short and long survival, respectively, while survival of patients with lung involvement was similar to those with bone metastases. A progressive worsening of survival was found from patients with one site of disease to patients with two or more. Patients with pleural effusion as the single site of disease showed a longer survival than those with lung or bone involvement (data not shown). Other variables such as poor performance status (PS), ER-negative tumour, short DFI were also found to be negatively correlated to overall survival. Elevated levels of CA 15-3 were significantly associated to poor life expectancy in overall patients. Patient stratification according to the disease extent identified at least three subgroups with separate survival curves (Figure 1). An inverse correlation between

© Cancer Research Campaign 1997

overall survival and both CA 15-3 levels and positivity and disease extent is shown in Table 2.

### Multivariate analysis for overall survival according to the Cox's model

Variables that demonstrated a prognostic significance in univariate analysis were further tested in a multiple regression analysis according to Cox's model. PS, ER status, the presence of visceral metastases, DFI and the disease extent confirmed their prognostic role (beta values: 0.39, –0.50, 0.43, –0.04, 0.41; $P < 0.001$). Conversely, the number of metastatic sites and CA 15-3 serum levels failed to demonstrate an independent role in predicting survival. Neither age nor menopausal status at relapse entered the model.

### DISCUSSION

This study confirmed the good sensitivity of CA 15-3 in advanced breast cancer patients. Serum marker values significantly correlated with the disease extent, and this strict relationship accounted for the negative prognosis of patients with elevated levels.

The CA 15-3 sensitivity in our series was close to that reported elsewhere (Safi et al, 1989; de Wit et al, 1992; Geraghty et al, 1992; Bombardieri et al, 1993; Hayes, 1993; Solétormos et al, 1993). We also confirmed the finding (Clark et al, 1987) that some initial prognostic characteristics of the primary tumour, such as ER status, remain to be significant for the development of subsequent metastases. As expected, other prognostic factors, such as visceral involvement, DFI, PS, and the number of disease sites have been confirmed to significantly influence overall survival. On the contrary, with respect to the aforementioned study (Clark et al, 1987), we were unable to demonstrate the negative impact of lymph node involvement at diagnosis, probably because 35% of data were not available.

As repeatedly observed, (Clark et al, 1987; Zinser et al, 1987; Koenders et al, 1992; Gregory et al, 1993), cases with visceral metastases and, particularly, liver involvement have been found to have the worst prognosis. However, the multivariate survival analysis clearly showed that the tumour load may also negatively influence the life expectancy, independently from the dominant site of disease.

The overall disease extent was assessed, in the present study, using the method firstly described by Swenerton et al (1979) and subsequently updated by Colomer et al (1989), who introduced information determined from the CT scan. Using the same criteria, we observed at least three patient subsets with divergent life expectancies, confirming the availability of this method for the overall tumour load determination.

The prognostic significance of CA 15-3 (Berruti et al, 1994) may be because of its relationship with disease extent, or, alternatively, the mucinous marker may reflect the state of cell differentiation and aggressiveness of the tumour (Saccani Jotti and Bombardieri, 1990). The increase of either marker levels or the frequency of supranormal values with the increase of tumour load suggest that CA 15-3 values reflect the number of breast cancer cells secreting the marker and its prognostic role is because of the strict relationship with disease extent. This hypothesis is supported by the finding that the prognostic influence of the marker is not confirmed in the multivariate Cox model in which disease extent and CA 15-3 were concomitantly tested.

The assessment of tumour load is time-consuming and often difficult in practice. In this study, in fact, disease extent was not evaluable in about 25% of the patients: 39 (12%) because of insufficient information and 41 (13%) because of non-measurable disease (pleural effusion, lymphangitis or ascites). The relationship between marker levels and disease extent supports the use of CA 15-3 in this respect, allowing a strict monitoring of tumour variation as a consequence of treatment administration (Tondini et al, 1988; Safi et al, 1989; Robertson et al, 1991). It should be noted, however, that the circulating marker values in patients with the same disease extent score were not homogeneous. We do not think that clearance mechanisms accounted for this discrepancy as patients with liver or renal impairment were not included. The recent finding of a spread presence of antibody against mucines in sera of patients with breast cancer may offer a possible explanation for the cases with high disease extent and low CA 15-3 levels (Gourevitch et al, 1995).

The usefulness of serial CA 15-3 evaluation in the follow-up of disease-free patients after mastectomy is debatable, mainly because it was found to be unable to detect the early stages of recurrence of disease. However, the relationship between CA 15-3 positivity at first relapse of disease and overall survival suggests that the marker could selectively detect patients with poor prognosis who may benefit from an early aggressive treatment.

### REFERENCES

Berruti A, Tampellini M, Torta M, Buniva T, Gorzegno G and Dogliotti L (1994) Prognostic value in predicting overall survival of two mucinous markers: CA 15-3 and CA 125 in breast cancer patients at first relapse of disease. *Eur J Cancer* **30A**: 2082–2084

Bombardieri E, Pizzichetta M, Veronesi P, Seregni E, Bogni A, Maffioli L, Saccani Jotti G, Bassetto MA, Zurrida S and Costa A (1993) CA 15-3 determination in patients with breast cancer: clinical utility for the detection of distant metastases. *Eur J Cancer* **29A**: 144–146

Chlebowski RT and Lillington LM (1994) A decade of breast cancer clinical investigation: results as reported in the program/proceedings of the American society of clinical oncology. *J Clin Oncol* **12**: 1789–1795

Clark GM, Sledge GW, Osborne CK and Mc Guire WL (1987) Survival from first recurrence: relative importance of prognostic factors in 1015 breast cancer patients. *J Clin Oncol* **5**: 55–61

Clavel M and Catimel G (1993) Breast cancer: chemotherapy in the treatment of advanced disease. *Eur J Cancer* **29A**: 598–604

Colomer R, Ruibal A and Salvador L (1989) Circulating tumour marker levels in advanced breast carcinoma correlate with the extent of metastatic disease. *Cancer* **64**: 1674–1681

de Wit R, Hoek FJ, Bakker PJ and Veenhof CH (1992) A comparison of CA-549 with CA 15-3 and MCA in patients with metastatic breast cancer. *Ann Oncol* **3**: 314–315

Dogliotti L, Faggiuolo R, Buniva T, Berruti A, Torta M and Tampellini M (1990) Serum CA 15-3 evaluation in breast cancer. *J Nucl Med All Sci* **34** (suppl.): 211–216

Dogliotti L, Berruti A, Buniva T, Zola P, Baù MG, Farris A, Sarobba MG, Bottini A, Arquati P, Deltetto F, Gosso P, Monzeglio C, Moro G, Sussio M and Perroni D (1996) Lonidamine significantly increases the activity of epirubicin in advanced breast cancer patients. Results from a multicenter prospective randomized trial. *J Clin Oncol* **14**: 1165–1172

Geraghty JG, Coveney EC, Sherry F, O'Higgins and Duffy MJ (1992) CA 15-3 in patients with locoregional and metastatic breast carcinoma. *Cancer* **70**: 2831–2834

Gion M, Cappelli G, Mione R, Pistorello M, Meo S, Vignati G, Fortunato A, Saracchini S, Biasoli R and Giulisano M (1994) Evaluation of critical differences of CEA and CA 15-3 levels in serial samples from patients operated for breast cancer. *Int J Biol Markers* **9**: 135–139

Gourevitch MM, Von Mensdorff-Pouilly S, Litvinov SV, Kenemans P, Van Kamp GJ, Verstraeten AA and Hilgers J (1995) Polymorphic epithelial mucin (MUC-1)-containing circulating immune complexes in carcinoma patients. *Br J Cancer* **72**: 934–938

© Cancer Research Campaign 1997

Gregory WM, Smith P, Richards MA, Twelves CJ, Knight RK and Rubens RD (1993) Chemotherapy of advanced breast cancer: outcome and prognostic factors. *Br J Cancer* **68**: 988–995

Hayes DF (1993) Tumor markers for breast cancer. *Ann Oncol* **4**: 807–819

Hayes DF, Henderson C and Shapiro C (1995) Treatment of metastatic breast cancer: present and future prospects. *Semin Oncol* **22**: 5–21

Henson DE, Ries L, Freedman LS and Carriaga M (1991) Relationship among outcome, stage of disease, and histological grade for 22,616 cases of breast cancer. *Cancer* **68**: 2142–2149

Koenders PG, Beex LV, Kloppenborg PW, Smals AG and Benraad TJ (1992) Human breast cancer: survival from first metastasis. *Breast Cancer Res Treat* **21**: 173–180

La Vecchia C, Negri E, Decarli A, Fasoli M and Cislaghi C (1990) Cancer mortality in Italy: an overview of age-specific and age-standardized trends from 1955 to 1984. *Tumori* **76**: 87–166

Nie NH, Hull CH and Jeakins JG (1988) *Statistical Package for the Social Sciences* **SPSS**: Chicago

Overmoyer BA (1995) Chemotherapeutic palliative approaches in the treatment of breast cancer. *Semin Oncol* **22**: 2–9

Robertson JFR, Pearson D, Price MR, Selby C, Blamey RW and Howell A (1991) Objective measurement of therapeutic response in breast cancer using tumour markers. *Br J Cancer* **64**: 757–763

Saccani Jotti G and Bombardieri E (1990) Circulating tumor markers in breast cancer. *Anticancer Res* **10**: 253–258

Safi F, Kohler I, Rottinger E, Suhr P and Beger HG (1989) Comparison of CA 15-3 and CEA in diagnosis and monitoring of breast cancer. *Int J Biol Markers* **4**: 207–214

Solétormos G, Nielsen D, Schioler V, Skovsgaard T, Winkel P, Mouridsen HT and Dombernowsky P (1993) A novel method for monitoring high-risk breast cancer with tumour markers: CA 15-3 compared to CEA and TPA. *Ann Oncol* **4**: 861–869

Swenerton KD, Legha SS, Smith T, Hortobagyi GN, Gehan EA, Yap Hwee-Y, Gutterman JU and Blumenschein GR (1979) Prognostic factors in metastatic breast cancer treated with combination chemotherapy. *Cancer Res* **39**: 1552–1562

Tondini C, Hayes DF, Gelman R, Henderson C and Kufe DW (1988) Comparison of CA 15-3 and carcinoembryonic antigen in monitoring the clinical course of patients with metastatic breast cancer. *Cancer Res* **48**: 4107–4112

Yadav GC, Rao A, Motawy MM, Safadi N and Ahmed MJ (1993) CA 15-3 with urinary calcium excretion is useful in the diagnosis and monitoring of bone metastases from breast cancer. *Int J Biol Markers* **8**: 208–214

Zinser JW, Hortobagyi GN, Buzdar AU, Smith T and Fraschini G (1987) Clinical course of breast cancer patients with liver metastases. *J Clin Oncol* **5**: 773–782

*© Cancer Research Campaign 1997*